# Comparison of diagnostic accuracy of three-dimensional transvaginal ultrasound and magnetic resonance imaging in the diagnosis of scar pregnancy

**DOI:** 10.12669/pjms.38.7.6288

**Published:** 2022

**Authors:** Zhulan Huang, Jinghua Liu, Zongyu Jing, Limei Lin, Xiaoyun Li

**Affiliations:** 1 Zhulan Huang, Department of Ultrasound, Longgang District Maternity & Child Healthcare Hospital of Shenzhen City, Shenzhen 518172, Guangdong Province, P.R. China; 2 Jinghua Liu, Department of Ultrasound, Longgang District Maternity & Child Healthcare Hospital of Shenzhen City, Shenzhen 518172, Guangdong Province, P.R. China; 3 Zongyu Jing, Department of Ultrasound, Longgang District Maternity & Child Healthcare Hospital of Shenzhen City, Shenzhen 518172, Guangdong Province, P.R. China; 4 Limei Lin, Department of Ultrasound, Longgang District Maternity & Child Healthcare Hospital of Shenzhen City, Shenzhen 518172, Guangdong Province, P.R. China; 5 Xiaoyun Li, Department of Ultrasound, Longgang District Maternity & Child Healthcare Hospital of Shenzhen City, Shenzhen 518172, Guangdong Province, P.R. China

**Keywords:** Three-dimensional transvaginal ultrasound, Magnetic resonance, Scar pregnancy, Diagnosis, Accuracy

## Abstract

**Objectives:**

To compare the accuracy of three-dimensional transvaginal ultrasound and magnetic resonance imaging in the diagnosis of scar pregnancy.

**Methods:**

The records of 54 patients with scar pregnancy, who underwent three-dimensional transvaginal ultrasound and magnetic resonance imaging (MRI), from June 2015 to November 2021 were reviewed. Surgery / histopathology of operative findings were analyzed as gold standard to compare the diagnosis of the two examination methods.

**Results:**

The detection rate of scar pregnancy by three-dimensional transvaginal ultrasound was 94.44%, which was not significantly different from MRI (96.30%, *P*>0.05). The accuracy, specificity and sensitivity of transvaginal three-dimensional ultrasonography in the diagnosis of scar pregnancy were 94.44%, 66.67% and 96.08%, respectively, and were not significantly different from MRI, 96.30%, 50.00% and 98.08% (*P*>0.05). The detection rates of yolk sac, embryo and heart tube pulsation by three-dimensional transvaginal ultrasound were higher than those detected by MRI (*P*<0.05). The detection rates of intrathecal hemorrhage, scar infiltration and uterine hematocele by MRI were significantly higher compared to three-dimensional transvaginal ultrasound (*P*<0.05). There was no significant difference between the two methods (*P*>0.05).

**Conclusion:**

Both three-dimensional transvaginal ultrasound and MRI have good diagnostic efficacy in the diagnosis of scar pregnancy. Detection rates of scar pregnancy diagnostic criteria differ between the two methods, and if necessary, the two methods can be used together, to further improve the diagnostic accuracy of scar pregnancy.

## INTRODUCTION

Scar pregnancy is a common, long-term, complication of a cesarean section, and refers to an ectopic pregnancy in which the gestational sac, fertilized egg and embryo are implanted at the scar of the uterine incision.^1^age-matched case-control study including 45 cases of PCSP patients after D&C was conducted between January 2013 and April 2018. For each case, 4 women who had been diagnosed with CSP and had the same age and same hospitalization period as the case group but no residual CSP tissue after D&C were selected as the controls (Control group, n = 180 In recent years, with the continuous improvement of cesarean section rates, the incidence of scar pregnancy has increased.^2^ While the cause of a scar pregnancy is not known, it is most likely due to poor healing of the abdominal scar after a cesarean section, resulting in the generation of microchannels and cracks between the scar and the endometrium.^3^ If the gestational sac appears in the cesarian scar, then further development of the embryo can lead to the invasion and growth of trophoblasts into the myometrium and cause a scar pregnancy. Furthermore, the extension of embryonic development can lead to trophoblast invasion.^4^

The scar is relatively weak and could cause either a partial or full rupture of the uterus, causing bleeding and endangering the lives of these patients.^5^ Early diagnosis of a scar pregnancy is very important. Three-dimensional transvaginal ultrasound, which is one of the more commonly used imaging methods, has almost no contraindications, and can clearly display the endometrium, clarify the location of pregnancy tissue, and judge the relationship between pregnancy tissue and the uterine scar. However, imaging of the surrounding tissue is not ideal.^6^ In recent years, magnetic resonance imaging (MRI) has been applied to the diagnosis of scar pregnancy. MRI has high resolution for soft tissue, can scan in multiple directions, display the endometrium, binding zone and grass-roots structure, and clarify the location of the scar pregnancy. However, MRI is expensive, with poor repeatability, and limited display of the fetal bud and fetal heart.^7^ Generally, the selection of either three-dimensional transvaginal ultrasound or MRI for diagnosis of scar pregnancy, is determined according to the patient’s condition or willingness. To further compare the diagnostic effects of these two methods, this study retrospectively analyzed the clinical data of 54 patients with scar pregnancy.

## METHODS

The records of 54 patients with scar pregnancy who underwent three-dimensional transvaginal ultrasound and MRI in Longgang District Maternity & Child Healthcare Hospital of Shenzhen City and Foshan Women and Children Hospital Affiliated to Southern Medical University from June 2015 to November 2021 were retrospectively selected.

### Inclusion Criteria:

- Clear history of cesarean section;
- Meets the diagnostic criteria of uterine scar pregnancy after cesarean section^8^ and has been confirmed by operation or pathology;
- Complete medical records.

### Exclusion Criteria:

- Combined with hysteromyoma, adenomyosis and other reproductive system diseases;
- Complicated with severe basic diseases, organ dysfunction and malignant tumors;
- Contraindications of three-dimensional transvaginal ultrasound or MRI;
- Cognitive and mental disorders.

This study has been approved by the medical ethics committee of Longgang District Maternity & Child Healthcare Hospital of Shenzhen City (Approval number: LGFYYXLLL-2021-001, Date: November 8th, 2021.

### Inspection method

Three-dimensional transvaginal ultrasound - the instrument used was a three-dimensional, color Doppler ultrasound (GE E10, USA). The RIC5-9 intracavitary volume probe was used with a frequency of 5 ~ 9MHz. The patient should have an empty bladder, and then instructed to lie on their back in the lithotomy position on the examination bed. A condom was placed on the ultrasonic probe, and gel applied before the probe was inserted into the vaginal fornix. First, a routine, two-dimensional ultrasound examination was carried out to observe the morphology of the uterus, and gestational sac. During the three-dimensional ultrasound, the volume and sampling frame were adjusted, the region of interest was located, and the tomography scanning occurred. The X, y and Z axes were used to scan the patient’s uterus and gestational sac.

MRI examination – A GE Optima MR360 1.5T scanner (GE, USA) was used. The patient was asked drink an appropriate amount of water prior to the examination to ensure their bladder was full. During the examination, the patient was instructed to lie on their back, and to breathe evenly. Imaging was completed through a series of scanning blocks, transverse, sagittal and coronal: Ax T2 Ideal, Ax T2 FRFSE shim, AxT1 FSE big fov, AxDWI b=800 shim scanning parameters. The layer of thickness was 5mm, the spacing was 1mm, and over 20 – 40 layers. The parameters of the other indexes were adjusted appropriately according to specific cases.

### Collection indicators

Detection of different types of scar pregnancy, including incision gestational sac type and mass type. Three-dimensional transvaginal ultrasound findings of gestational sac scar pregnancy:^9^ while blood signals were detected by 3D power Doppler ultrasound. Endometrial volPak J Med Sci September - October 2022 Vol. 38 No. 7 www.pjms.org.pk 1745 ume (EV10 The gestational sac was implanted at the original surgical scar, some yolk sac, germ or cardiac tube pulsation can be seen, the echo of muscle layer near the gestational sac was relatively uniform, and circular blood flow signal could be seen.

### MRI findings of gestational sac scar pregnancy:^11^,^12^

The gestational sac was attached to the surgical scar, and a round, long T1 and T2 signal shadow could be seen. Three-dimensional transvaginal ultrasound findings of mass scar pregnancy: there was no gestational sac echo in the cervical canal or uterine cavity, the gestational sac showed an uneven echo with a mixed mass, the boundary with the uterine myometrium was blurred, and blood flow signals appeared in and around the mass.

### MRI findings of mass scar pregnancy:

The gestational sac protruded into the scar, showing mixed T1WI and T2WI signals.

### Diagnostic accuracy, specificity and sensitivity. Formula

a is true positive, b is false positive, c is false negative, d is true negative, accuracy=(a+d) / total number of cases, specificity=d/(b+d), sensitivity=a/(a+c).

### The detection of scar pregnancy signs

gestational sac, yolk sac, embryo, cardiac pulsation, intrathecal hemorrhage, local scar infiltration and uterine hematocele.

### Statistical analysis

SPSS22.0 was used for data processing, [n (%)] was used to represent non grade count data, and the test method was *χ^2^*. Measurement data was represented by (*x̅*±*s*), and a *t*-test was performed. Data was considered statistically significant when *P*< 0.05.

## RESULTS

A total of 54 patients met the inclusion criteria. Their ages ranged from 22 to 36 years, with an average of 29.12±4.57 years. The duration of pregnancy ranged from five to twelve weeks, with an average of 8.50±3.23 weeks. The number of cesarean sections was once in 34 cases and ≥ twice in 20 cases.

The detection rate of scar pregnancy using three-dimensional transvaginal ultrasound was not statistically significant when compared to MRI (94.44% *vs*. 96.30%; *P*>0.05) (Table-I). The accuracy, specificity and sensitivity of transvaginal three-dimensional ultrasonography in the diagnosis of scar pregnancy were not significantly different from MRI (94.44%, 66.67% and 96.08% *vs*. 96.30%, 50.00% and 98.08%; *P*>0.05) (Table-II). The detection rate of the yolk sac, embryo and heart tube pulsation by three-dimensional transvaginal ultrasound was higher than that measured with MRI (*P*<0.05). The detection rate of intrathecal hemorrhage, scar infiltration and uterine hematocele using MRI was higher than that measured by three-dimensional transvaginal ultrasound (*P*<0.05). There was no significant difference in the detection rate of gestational sac between the two methods (*P*>0.05) (Table-III).

**Table I:** Comparison of detection rates of different types of scar pregnancy [n (%)].

Inspection Method	n	Gestational sac type	Bulk type	Coincidence rate
Surgery or pathology	54	32 (59.26)	22 (40.74)	-
Transvaginal three-dimensional ultrasound	54	30 (55.56)	21 (38.89)	51 (94.44)
Magnetic Resonance Imaging (MRI)	54	31 (57.41)	21 (38.89)	52 (96.30)
χ^2^	-		-	0.210
P	-	-	-	0.647

**Table II:** Comparison of the diagnostic efficacy of transvaginal three-dimensional ultrasound and MRI.

Inspection Method	n	True positive (n)	False positive (n)	False negative (n)	True negative (n)	Accuracy (%)	Specificity (%)	Sensitivity (%)
Transvaginal three-dimensional ultrasound	54	49	1	2	2	94.44	66.67	96.082
Magnetic Resonance Imaging (MRI)	54	51	1	1	1	96.30	50	98.083
χ^2^	-	-	-	-	-	0.210	3.086	0.343
P	-	-	-	-	-	0.647	0.079	0.558

**Table III:** Comparison of the detection of scar pregnancy signs between transvaginal three-dimensional ultrasound and MRI [n (%)].

Inspection Method	n	Gestational sac	Yolk sac	Germ	Heart rate	Gestational sac bleeding	Local scar infiltration	Uterine hemorrhage
Surgery or pathology	54	50	26	15	13	22	30	26
Transvaginal three-dimensional ultrasound	54	47	24	14	12	16	22	20
Magnetic Resonance Imaging (MRI)	54	49	18	9	7	21	28	25
χ^2^	-	1.042	4.457	4.658	4.887	4.247	4.320	4.127
P	-	0.307	0.035	0.031	0.027	0.039	0.038	0.042

## DISCUSSION

The results from this study highlight the similarities in the detection rate, diagnostic accuracy, specificity and sensitivity of scar pregnancy diagnosis between three-dimensional transvaginal ultrasound and MRI (*P*>0.05). Both methods demonstrated good diagnostic efficacy and may be used in combination to enhance diagnosis.

Previous research by Chong Y *et al*^13^ also found that both three-dimensional transvaginal ultrasound and MRI showed no significant differences in detection rate and diagnostic accuracy, of scar pregnancy. Three-dimensional transvaginal ultrasound uses an ultrasonic probe to observe the uterus and its surrounding tissue at a close distance with a high resolution. During pregnancy, the echo of the gestational sac, blood flow and muscle wall thickness are important measurements.^14^ Three-dimensional transvaginal ultrasound can image the pregnancy tissue, uterus and its surrounding tissue from different angles, and display the position, size and shape of the pregnancy sac, so as to accurately detect different types of scar pregnancy. While MRI has a very high resolution for soft tissue, and can accurately identify the myometrium, decidua and serosa, and multi-directional and multi sequence imaging. MRI can more intuitively display the position, external and internal specific results of the pregnancy sac, to accurately detect different types of scar pregnancy.^15^ There is a certain complexity in the diagnosis and treatment of scar pregnancy in its early stage. To ensure the appropriate clinical intervention is chosen, it is necessary to identify the relationship of the pregnancy sac with surrounding tissues and whether there are signs of bleeding.^1^who needed a uterine artery embolization (UAE^6^ In the clinic, because the specific symptoms of scar pregnancy may not be obvious, it is easy to be confused by uterine hematocele, trophoblast, cystic abortion and incomplete abortion, resulting in misdiagnosis and missed diagnosis.^17^ In this study, the detection rates of the yolk sac, embryo and cardiac pulsation by three-dimensional transvaginal ultrasound were higher than those by MRI (*P*<0.05). While the detection rates of intrathecal hemorrhage, scar infiltration and uterine hematocele by MRI were higher than those by three-dimensional transvaginal ultrasound (*P*<0.05), with no significant difference between the two methods (*P*>0.05). These results are consistent with those of Jain *et al*.^18^ Transvaginal ultrasound has the advantage of convenience and high repeatability. This method can display the size, position and blood flow of the gestational sac, and dynamically observe the fetal bud and fetal heart rate. However, transvaginal ultrasound has difficulty displaying the relationship between the scar pregnancy tissue and adjacent tissues. Furthermore, it is impossible to accurately discern the distance between the gestational sac and the uterine serosa or the thickness of the uterine myometrium at the gestational sac.^1^suggesting that prelabor Cesarean section (CS^9^ In contrast, MRI can clearly show the relationship between the gestational sac and the scar, the depth of infiltration of the gestational sac into the surrounding tissues and measure the size of the focus and observe any bleeding. However, MRI is slightly insufficient in the display of fetal heart rate and fetal bud.^20^ Therefore, in the diagnosis of scar pregnancy, three-dimensional transvaginal ultrasound and MRI have their own advantages, and the combination of these two examination methods may be advantageous in the diagnosis of scar pregnancy. Specifically, when three-dimensional transvaginal ultrasound is unable to diagnose scar pregnancy independently, MRI may improve the diagnostic accuracy and provide reference for clinical treatment.

### Limitation of the study

The sample size was relatively small, with only 54 patients receiving either examination method within two hospital, over six years. Additionally, imaging techniques, such as ultrasound and MRI, do have a relatively high subjectively, which may make the conclusions one-sided and limited.

## CONCLUSION

In the diagnosis of scar pregnancy, three-dimensional transvaginal ultrasound and MRI both demonstrate good diagnostic efficiency. Both methods have their own advantages, and if necessary, can be combined to enhance the diagnostic accuracy of detecting scar pregnancy.

### Authors’ contributions:

**ZH:** Conceived and designed the study. **JL, ZJ, LL & XL:** Collected the data and performed the analysis.**ZH:** Was involved in the writing of the manuscript and is responsible for the integrity of the study. All authors have read and approved the final manuscript.
